# Semiquantitative SSTR PET metrics show molecular–morphologic discordance after 177Lu-DOTATATE in gastroenteropancreatic neuroendocrine neoplasms: an exploratory paired target-lesion analysis

**DOI:** 10.1007/s12149-026-02231-y

**Published:** 2026-05-18

**Authors:** Agostino Chiaravalloti, Martina Egidi, Antonio D’Agostini, Luca Filippi, Elizabeth Katherine Anna Triumbari, Oreste Bagni, Orazio Schillaci

**Affiliations:** 1 Department of Biomedicine and Prevention, University Tor Vergata, Via Montpellier 1, Rome, 00133 IT Italy; 2 IRCCS Neuromed, Pozzilli, Italy; 3 Santa Maria Goretti Hospital, Latina, Italy

**Keywords:** Gastroenteropancreatic neuroendocrine neoplasms, Peptide receptor radionuclide therapy, ^177Lu-DOTATATE, Somatostatin receptor PET, Treatment response

## Abstract

**Objective:**

To assess whether simple paired semiquantitative somatostatin receptor (SSTR) PET metrics identify treatment-related changes after 177Lu-DOTATATE in patients with gastroenteropancreatic neuroendocrine neoplasms (GEP-NENs) and to explore their relationship with single-lesion size measured on contrast-enhanced CT in an exploratory paired target-lesion framework.

**Methods:**

We retrospectively evaluated 29 consecutive patients with GEP-NENs who completed four cycles of 177Lu-DOTATATE and underwent baseline and post-treatment 68Ga-DOTA-peptide PET/CT. Median age at treatment initiation was 64.5 years, 18/29 patients were male, 15 had a pancreatic primary, 13 an intestinal primary, and 1 a pancreatic/duodenal primary of uncertain origin. Most tumors were grade 2 (17/29), and liver metastases were present in 26/29 patients. For each patient, semiquantitative measurements were extracted from the target lesion recorded in the institutional database at baseline and post-treatment assessment. SUVmax, SUVmean, target lesion size, Krenning score, tumor-to-liver ratio, and tumor-to-blood ratio were compared between baseline and post-treatment imaging. Exploratory molecular response was defined as a reduction of at least 30% in semiquantitative PET parameters, whereas exploratory morphologic change was defined as a reduction of at least 20% in lesion size measured on contrast-enhanced CT. Baseline PET/CT was typically performed within 4–6 weeks before PRRT initiation, and post-treatment PET/CT approximately 3 months after PRRT completion according to institutional routine practice.

**Results:**

Median target lesion SUVmax decreased from 52.22 to 29.73 (*p* = 0.0007), and median SUVmean decreased from 11.44 to 7.25 (*p* = 0.023). Median target lesion size changed from 2.80 to 2.49 cm and was not significantly different (*p* = 0.168). Tumor-to-liver ratio decreased from 9.38 to 5.31 (*p* < 0.001), and tumor-to-blood ratio from 45.96 to 22.57 (*p* < 0.001). Krenning score showed a modest but significant shift despite an unchanged median value of 4 (*p* = 0.0049). Using predefined exploratory thresholds, molecular response based on tumor-to-blood ratio was observed in 20/29 patients, whereas exploratory contrast-enhanced CT-based size reduction was observed in 9/29; 14 of 20 patients with molecular response showed no concomitant size reduction. Among 24 patients with evaluable final imaging response, a tumor-to-blood ratio reduction of at least 30% was observed in 10/11 patients with complete or partial response and in 7/13 with stable or progressive disease.

**Conclusions:**

In this exploratory paired target-lesion analysis, semiquantitative SSTR PET metrics showed significant treatment-related changes after 177Lu-DOTATATE, whereas paired single-lesion size changes on contrast-enhanced CT were modest. These findings support the presence of molecular–morphologic discordance after PRRT and suggest that normalized PET-derived metrics deserve further investigation as practical tools for post-treatment assessment in GEP-NENs.

## Introduction

Gastroenteropancreatic neuroendocrine neoplasms (GEP-NENs) are a biologically heterogeneous group of tumors in which somatostatin receptor (SSTR) expression provides the rationale for both receptor imaging and peptide receptor radionuclide therapy (PRRT). Over the last few years, ^177Lu-DOTATATE has become a mainstay in the treatment of SSTR-positive disease, although the optimal timing of PRRT within the therapeutic sequence remains debated and a validated predictive biomarker is still lacking [1, 2].

Despite the established clinical role of PRRT, response assessment remains problematic. As emphasized in a dedicated review on post-PRRT evaluation, no consensus has yet been reached regarding the best methodology and timing for response assessment, partly because conventional morphologic criteria may be suboptimal in slowly growing tumors and may underestimate treatment effect in the presence of therapy-related necrotic changes [3]. This limitation has stimulated increasing interest in SSTR PET-derived biomarkers. Several studies have shown that quantitative SSTR PET parameters may provide clinically relevant information before or during PRRT. In a retrospective series of 55 metastatic NET patients treated with ^177Lu-DOTATATE, Sharma et al. reported RECIST responses in 47 evaluable patients, with partial response, stable disease, and progressive disease observed in 28%, 60%, and 13% of cases, respectively; notably, objective response after PRRT was associated with markedly different progression-free survival (71.8 months for partial response, 29.1 months for stable disease, and 9.7 months for progressive disease), while baseline single-lesion SUVmax predicted both response and progression-free survival, with a cutoff of 13.0, and correlated with SSTR2 expression (Spearman’s rho = 0.69) [4]. More recently, Hotta et al. analyzed 40 NET patients who completed four cycles of ^177Lu-DOTATATE and found that 14/40 patients (35%) achieved RECIST partial response, whereas progression occurred in 17/40 (42.5%); higher baseline whole-body SUVmean and hottest-lesion SUVmax were associated with better radiologic response (median WB-SUVmean 28.0 vs. 14.6, *p* = 0.015; median H-SUVmax 68.4 vs. 35.0, *p* = 0.005), while low whole-body SUVmean and the presence of SSTR-PET-negative lesions were associated with shorter progression-free survival, with adjusted hazard ratios of 6.22 and 3.80, respectively [5].

Additional evidence suggests that normalized and dynamic quantitative PET parameters may be particularly informative. In the prospective phase II LUMEN study, Karfis et al. evaluated 37 progressive GEP-NET patients and observed a partial response in 11/37 (30%), with a median progression-free survival of 28 months; importantly, patients showing a decrease in SSTR tumor volume greater than 10% after the first PRRT cycle had significantly longer progression-free survival than those without such decrease (51.3 vs. 22.8 months; hazard ratio 0.35) [6]. Along the same line, Aouf et al. performed a lesion-based analysis of 71 lesions in 32 metastatic GEP-NET patients and found that baseline SUVmax and SUVmean were poorly correlated with lesion-based molecular response, whereas tumor-to-liver ratios were significantly associated with functional response (SUVmaxT/L: *p* = 0.004, *r* = 0.434; SUVmeanT/L: *p* = 0.011, *r* = 0.412) [7]. Taken together, these studies support the clinical relevance of receptor-based quantitative imaging in PRRT, but the field remains methodologically fragmented. Most available reports have focused on baseline prognostic stratification, whole-body volumetric metrics, early cycle-based assessment, or lesion-based predictive analyses using relatively complex workflows [4–7]. In contrast, fewer data are available on simple paired post-treatment semiquantitative SSTR PET metrics directly compared with concomitant morphologic lesion size changes in routine clinical practice. This distinction is clinically relevant, because treatment-related receptor changes may occur without parallel tumor shrinkage, potentially leading to underestimation of benefit when post-PRRT evaluation relies mainly on morphologic criteria. On this basis, the aim of the present study was to investigate whether simple paired semiquantitative SSTR PET parameters, including normalized uptake ratios, identify treatment-related changes after 177Lu-DOTATATE and how these changes relate to conventional morphologic lesion size in a real-world cohort of patients with GEP-NENs.

## Materials and methods

### Study design and patient population

This was a retrospective, single-center study conducted at Santa Maria Goretti Hospital, Latina, Italy. We reviewed our institutional database of patients with gastroenteropancreatic neuroendocrine neoplasms (GEP-NENs) treated with peptide receptor radionuclide therapy (PRRT) using ^177Lu-DOTATATE. Consecutive patients were eligible for the present analysis if they had histologically proven GEP-NEN, completed four cycles of PRRT, and underwent both baseline and post-treatment somatostatin receptor (SSTR) PET/CT with ^68Ga-DOTA-TOC, with extractable semiquantitative imaging variables at both time points.

Patients without paired pre- and post-treatment PET-derived data suitable for quantitative comparison were excluded. Clinical variables extracted from the database included age at PRRT initiation, sex, primary tumor site, tumor grade, metastatic distribution, previous surgery, previous systemic treatments, somatostatin analog therapy, selected laboratory parameters, and follow-up data. Final post-treatment response category, when available in the clinical database, was recorded as complete response (CR), partial response (PR), stable disease (SD), or progressive disease (PD). These categories were database-derived clinico-radiologic assessments, typically assigned approximately 3 months after completion of PRRT, with subsequent periodic follow-up evaluations.

### PRRT protocol

PRRT was administered at Santa Maria Goretti Hospital according to routine institutional clinical practice, in line with published procedure standards for ^177Lu-DOTATATE therapy. In brief, treatment consisted of four cycles of ^177Lu-DOTATATE administered at standard intervals, with concomitant amino acid infusion for renal protection and appropriate supportive care. Patient selection for treatment was based on SSTR-positive disease documented on baseline receptor imaging, with tumor uptake exceeding background liver uptake, consistent with current practice standards. Long-acting somatostatin analog therapy was managed according to standard recommendations for PRRT scheduling [8].

### ^68Ga-DOTA-TOC PET/CT

Baseline and post-treatment receptor imaging was performed with ^68Ga-DOTA-TOC PET/CT at Santa Maria Goretti Hospital as part of routine clinical care. PET/CT acquisition and interpretation were performed according to the local institutional protocol, consistent with published EANM procedural recommendations for imaging of neuroendocrine neoplasms with ^68Ga-DOTA-conjugated peptides. According to these recommendations, PET acquisition is generally performed after intravenous administration of approximately 100–200 MBq of tracer, with image acquisition typically obtained 60–90 min after injection for ^68Ga-DOTA-TOC, covering the body from the head to the mid-thigh, with iterative reconstruction and standard corrections. Patients were studied under standard preparation conditions recommended for SSTR PET/CT [9].

For the present study, the baseline scan was defined as the last available pre-PRRT 68Ga-DOTA-TOC PET/CT, whereas the post-treatment scan was defined as the PET/CT performed after completion of PRRT and used for routine response assessment. According to institutional routine practice, baseline PET/CT was typically performed within 4–6 weeks before PRRT initiation, whereas post-treatment PET/CT was generally performed approximately 3 months after completion of PRRT.

### Image analysis

This study was designed as an exploratory paired target-lesion analysis based on the institutional database. According to the institutional imaging workflow, the target lesion was prospectively defined at baseline as the lesion with the highest SUVmax, and the same baseline target lesion was then reassessed after completion of PRRT. No independent reselection of the hottest lesion was performed at post-treatment assessment. In this retrospective study, semiquantitative values were extracted from the institutional database rather than from a centralized rereading workflow. Because lesion descriptors in the database were not fully standardized across time points, formal independent retrospective verification of lesion matching was not possible in all cases. This limitation therefore concerns retrospective auditability of the recorded target lesion rather than the original clinical lesion-selection procedure.

Because this was a retrospective database-based study, the analysis relied on semiquantitative measurements already available in the institutional dataset rather than on a fully blinded central image rereading. For each target lesion, the following variables were collected:

- lesion SUVmax;
- lesion SUVmean;
- maximum lesion diameter;
- Krenning score.

Lesion SUVmean was obtained from a 3-dimensional volume of interest automatically adapted to a 70% isocontour threshold, in line with previously published 68Ga-DOTA-TOC PET/CT follow-up methodology in PRRT-treated NET patients [10].

In addition, uptake values of reference tissues available in the database were collected, including liver, blood pool, spleen, and kidneys. Based on these variables, the following normalized semiquantitative ratios were calculated:

- tumor-to-liver ratio (TLRmax) = target lesion SUVmax / liver SUVmean.
- tumor-to-blood ratio (TBRmax) = target lesion SUVmax / blood-pool SUVmean.
- tumor-to-spleen ratio (TSRmax) = target lesion SUVmax / spleen SUVmean.
- tumor-to-kidney ratio (TKRmax) = target lesion SUVmax / kidney SUVmean.

Target lesion size was defined as the maximum diameter of the baseline target lesion measured on the contrast-enhanced CT component of PET/CT and reassessed on the post-treatment study. Because formal independent retrospective verification of lesion matching was not possible in all cases from the database, size-based changes were used as an exploratory morphologic comparator and were not intended as formal RECIST 1.1 assessment.

### Study endpoints

The primary aim of the study was to assess whether simple paired semiquantitative SSTR PET parameters identified treatment-related changes after 177Lu-DOTATATE in an exploratory paired target-lesion framework and to compare these changes with single-lesion size measured on contrast-enhanced CT.

The primary imaging endpoint was the paired pre- versus post-treatment change in TLRmax. Secondary imaging endpoints included paired changes in TBRmax, SUVmax, SUVmean, target lesion size, Krenning score, TSRmax, and TKRmax.

As an exploratory analysis, molecular–morphologic discordance was evaluated using predefined pragmatic thresholds: exploratory molecular response was defined as a reduction of at least 30% in a semiquantitative PET parameter, whereas exploratory morphologic change was defined as a reduction of at least 20% in target lesion diameter measured on contrast-enhanced CT. These thresholds were used for descriptive and hypothesis-generating purposes only and do not represent validated RECIST 1.1 criteria.

A further exploratory analysis was performed in patients with an available final post-treatment response category in the clinical database. For this purpose, patients were dichotomized as:

- responders: CR or PR;
- non-responders: SD or PD.

Because of the retrospective nature of the study, the database-derived nature of the clinico-radiologic response categories, and the absence of a formal RECIST 1.1 annotation for this variable, this analysis was considered exploratory.

### Statistical analysis

Continuous variables were expressed as median and interquartile range (IQR), whereas categorical variables were reported as number and percentage. Paired comparisons between baseline and post-treatment imaging variables were performed using the Wilcoxon signed-rank test. Percent change for each imaging variable was calculated as:

$$\:{\Delta\:}\mathrm{\%}=\frac{\mathrm{post-treatment\:value}-\mathrm{pre-treatment\:value}}{\mathrm{pre-treatment\:va}\mathrm{lue}}\times\:100$$

Associations between exploratory dichotomized imaging variables and final response categories were assessed descriptively and, when appropriate, using Fisher’s exact test. All statistical tests were two-sided, and a p value < 0.05 was considered statistically significant. Missing data were not imputed, and all analyses were performed on available cases.

### Ethics

The study was retrospective and was approved by the local Ethics Committee. Given the retrospective design and the use of anonymized clinical and imaging data, the requirement for written informed consent was waived. The study was conducted in accordance with the ethical standards of the institutional research committee and with the 1964 Declaration of Helsinki and its later amendments.

## Results

### Patient characteristics

Baseline patient characteristics are summarized in Table 1. A total of 29 patients met the inclusion criteria and were included in the paired imaging analysis. Median age at PRRT initiation was 64.5 years (IQR 57.8–70.4), and 18/29 patients (62.1%) were male. The primary tumor site was pancreatic in 15/29 patients (51.7%), intestinal in 13/29 (44.8%), and pancreatic/duodenal of uncertain origin in 1/29 (3.4%). Tumor grade was G1 in 12/29 patients (41.4%) and G2 in 17/29 (58.6%). Liver metastases were present in 26/29 patients (89.7%). Previous surgery had been performed in 18/29 patients (62.1%), while 28/29 patients (96.6%) had received somatostatin analog therapy before PRRT.

**Table 1:** Baseline patient characteristics

Characteristic	Value
Patients, n	29
Age at PRRT initiation, years	64.5 (57.8–70.4)
Male sex, n (%)	18 (62.1)
Pancreatic primary, n (%)	15 (51.7)
Intestinal primary, n (%)	13 (44.8)
Pancreatic/duodenal uncertain primary, n (%)	1 (3.4)
Grade 1, n (%)	12 (41.4)
Grade 2, n (%)	17 (58.6)
Ki-67, %	4.5 (1.8–6.2)
Liver metastases, n (%)	26 (89.7)
Lymph node metastases, n (%)	9 (31.0)
Peritoneal metastases, n (%)	3 (10.3)
Bone metastases, n (%)	2 (6.9)
Previous surgery, n (%)	18 (62.1)
Previous chemotherapy/radiotherapy, n (%)	3 (10.3)
Previous targeted therapy, n (%)	6 (20.7)
Previous SSA therapy, n (%)	28 (96.6)

Continuous variables are reported as median (interquartile range); categorical variables as number (percentage)

Final post-treatment response categorization extracted from the clinical database was available in 24/29 patients (82.8%). Among evaluable patients, complete response was documented in 2/24 patients (8.3%), partial response in 9/24 (37.5%), stable disease in 8/24 (33.3%), and progressive disease in 5/24 (20.8%).

### Paired imaging findings

**Fig. 1:**
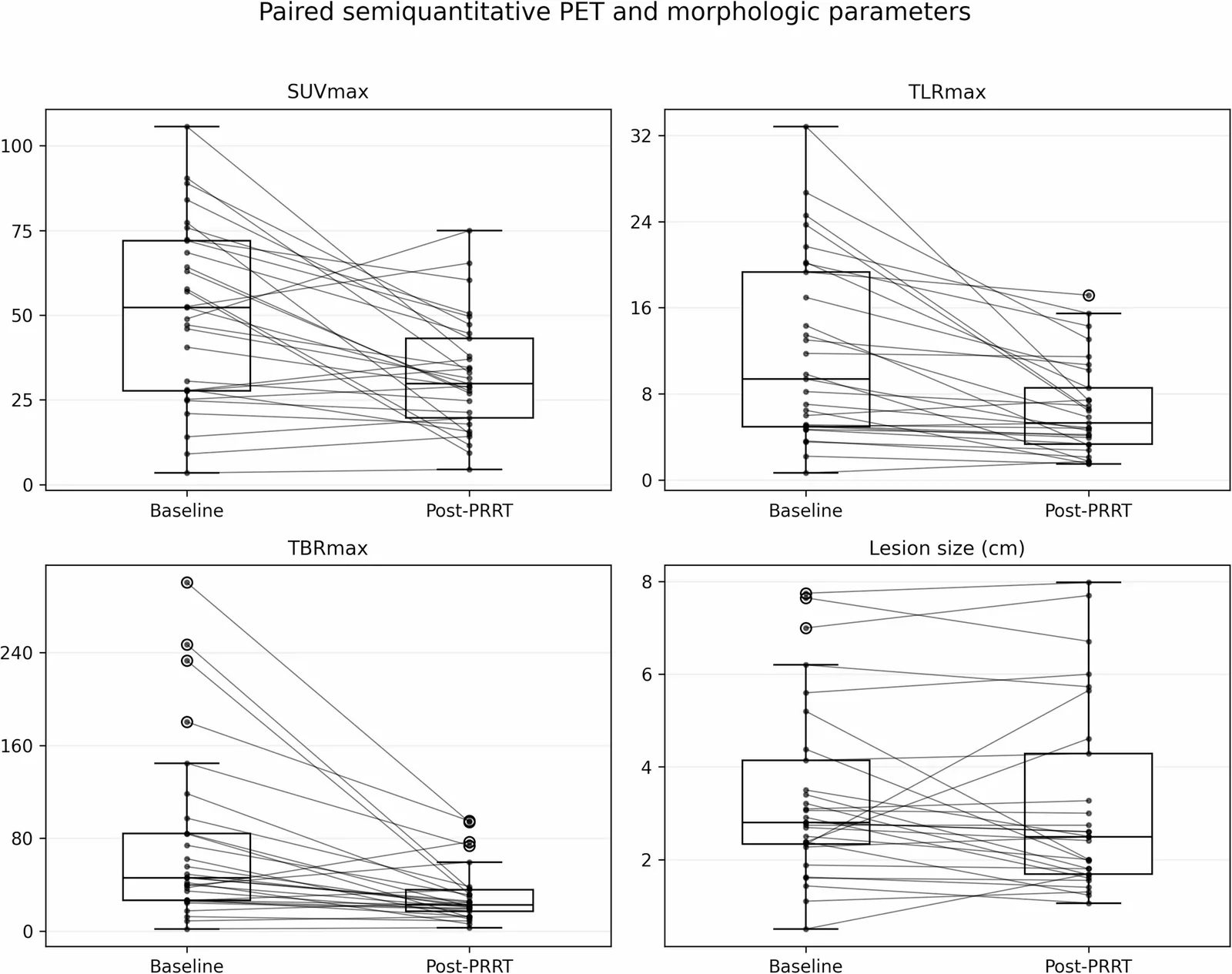
Paired boxplots of SUVmax, TLRmax, TBRmax, and contrast-enhanced CT lesion size before and after PRRT. Individual lines connect paired baseline and post-treatment values for each patient

Paired semiquantitative imaging findings are summarized in Table 2. In the paired target-lesion analysis, a significant reduction in receptor-based PET parameters was observed after completion of ^177Lu-DOTATATE. Median target lesion SUVmax decreased from 52.22 (IQR 27.71–71.99) at baseline to 29.73 (IQR 19.64–43.15) post-treatment (*p* < 0.001), whereas median SUVmean decreased from 11.44 (IQR 6.63–14.71) to 7.25 (IQR 4.71–9.15) (*p* = 0.023). By contrast, the maximum diameter of the target lesion measured on the contrast-enhanced CT component showed only a modest and statistically non-significant reduction, changing from 2.80 cm (IQR 2.33–4.14) at baseline to 2.49 cm (IQR 1.69–4.29) after treatment (*p* = 0.168). Normalized PET-derived ratios showed an even more marked treatment-related decline. Median TLRmax decreased from 9.38 (IQR 4.96–19.30) to 5.31 (IQR 3.35–8.56) (*p* < 0.001), while median TBRmax decreased from 45.96 (IQR 26.39–84.23) to 22.57 (IQR 17.17–35.58) (*p* < 0.001). A significant reduction was also observed for TSRmax, from 2.92 (IQR 1.46–5.37) to 1.81 (IQR 1.05–2.89) (*p* = 0.001), and for TKRmax, from 6.83 (IQR 2.98–9.93) to 4.55 (IQR 2.68–5.67) (*p* < 0.001). Krenning score was available at both time points in 28 patients and showed a modest but significant shift despite an unchanged median value of 4 (baseline 4 [IQR 4–4] vs. post-treatment 4 [IQR 3–4], *p* = 0.005). As illustrated in Fig. 1, semiquantitative PET-derived variables showed larger paired changes than single-lesion size measured on contrast-enhanced CT in this exploratory paired target-lesion framework.

**Table 2:** Paired imaging parameters before and after PRRT

Parameter	Pairs (*n*)	Baseline median (IQR)	Post-treatment median (IQR)	*p* value
SUVmax	29	52.22 (27.71–71.99)	29.73 (19.64–43.15)	< 0.001
SUVmean	29	11.44 (6.63–14.71)	7.25 (4.71–9.15)	0.023
Contrast-enhanced CT lesion size (cm)	29	2.80 (2.33–4.14)	2.49 (1.69–4.29)	0.168
TLRmax	29	9.38 (4.96–19.30)	5.31 (3.35–8.56)	< 0.001
TBRmax	29	45.96 (26.39–84.23)	22.57 (17.17–35.58)	< 0.001
TSRmax	28	2.92 (1.46–5.37)	1.81 (1.05–2.89)	0.001
TKRmax	29	6.83 (2.98–9.93)	4.55 (2.68–5.67)	< 0.001
Krenning score	28	4 (4–4)	4 (3–4)	0.005

Paired comparisons were performed with the Wilcoxon signed-rank test

### Exploratory molecular–morphologic discordance

**Fig. 2:**
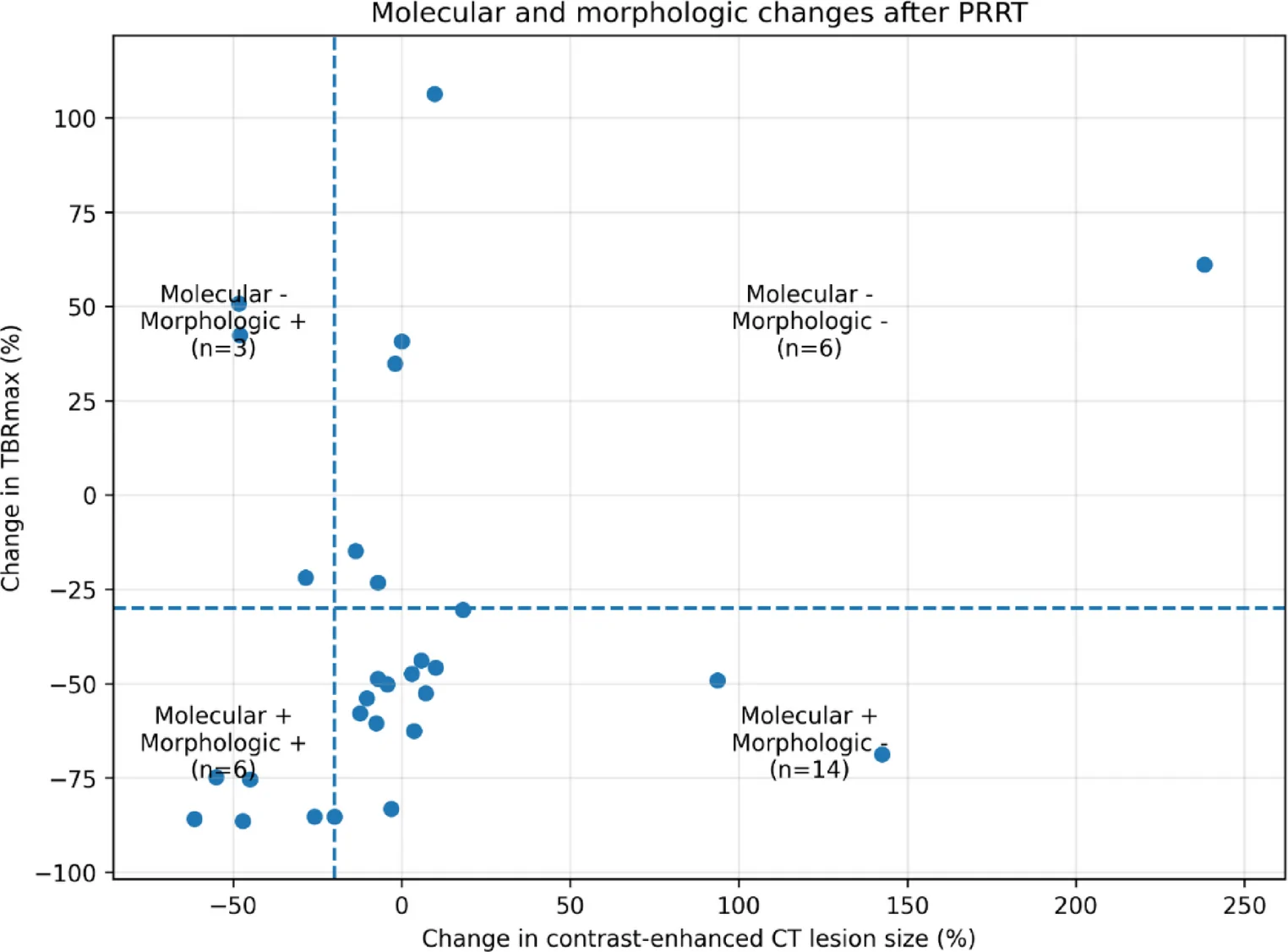
Relationship between molecular and morphologic changes after PRRT. Scatter plot showing percentage change in TBRmax versus percentage change in contrast-enhanced CT target lesion size for each patient. The horizontal dashed line indicates the exploratory threshold for molecular response (− 30% TBRmax), and the vertical dashed line indicates the exploratory threshold for morphologic size reduction (− 20%). This representation highlights the frequency of molecular improvement in the absence of concomitant lesion shrinkage

Using the predefined exploratory thresholds, a molecular response based on a TBRmax reduction of at least 30% was observed in 20/29 patients (69.0%), whereas an exploratory contrast-enhanced CT-based size reduction of at least 20% was observed in only 9/29 patients (31.0%). Among the 20 patients classified as molecular responders according to TBRmax, 14 (70.0%) showed no concomitant size reduction according to the predefined CT-based threshold. When TLRmax reduction of at least 30% was used as the molecular response criterion, 14/29 patients (48.3%) met the definition of molecular response; among them, 8/14 (57.1%) did not show a concomitant contrast-enhanced CT-based size reduction. The relationship between molecular and morphologic changes is shown in Fig. 2. Most patients fell into the lower-right quadrant, indicating a reduction in TBRmax without a concomitant contrast-enhanced CT-based size reduction. Specifically, 14/29 patients showed molecular improvement according to the predefined TBRmax threshold in the absence of concomitant morphologic shrinkage, whereas only 6/29 showed both molecular and morphologic response. This distribution further supports the presence of molecular–morphologic discordance after PRRT.

### Exploratory association with final response category

Among the 24 patients with available final post-treatment response categorization, complete or partial response was observed in 11 patients, whereas stable or progressive disease was observed in 13. A TBRmax reduction of at least 30% was observed in 10/11 patients (90.9%) with complete or partial response and in 7/13 patients (53.8%) with stable or progressive disease, corresponding to a numerical trend but not a statistically significant association in this small cohort (*p* = 0.078, Fisher’s exact test).

By comparison, an exploratory contrast-enhanced CT-based size reduction of at least 20% was observed in 4/11 patients (36.4%) with complete or partial response and in 3/13 patients (23.1%) with stable or progressive disease (*p* = 0.659). Overall, the molecular criterion showed a stronger numerical association with favorable final response categories than single-lesion size, although this finding should be considered exploratory.

## Discussion

In this retrospective single-center study, simple paired semiquantitative SSTR PET metrics showed significant treatment-related changes after 177Lu-DOTATATE, whereas paired single-lesion size changes measured on contrast-enhanced CT were modest and did not reach statistical significance. In particular, SUVmax, SUVmean, TLRmax, and TBRmax all decreased significantly after PRRT, whereas the reduction in target lesion diameter did not reach statistical significance. Moreover, a substantial proportion of patients with a marked molecular improvement did not show a parallel size reduction, supporting the presence of molecular–morphologic discordance after PRRT in routine clinical practice. This finding is biologically plausible in GEP-NENs, in which treatment benefit may not immediately translate into tumor shrinkage and in which post-PRRT response assessment remains methodologically challenging [1–3]. Our findings fit into an expanding literature showing that receptor-based imaging captures clinically relevant information beyond conventional lesion size, although most available studies have focused on baseline prognostic stratification or whole-body tumor burden rather than on simple paired post-treatment metrics. In the study by Sharma et al., baseline single-lesion SUVmax predicted both objective response and progression-free survival after PRRT [4], whereas Hotta et al. showed that higher baseline whole-body SUVmean and hottest-lesion SUVmax were associated with better radiologic response and longer progression-free survival after ^177Lu-DOTATATE [5]. Likewise, Toriihara et al. reported in 92 patients with well-differentiated NETs that volumetric parameters derived from ^68Ga-DOTATATE PET/CT, rather than hottest-lesion SUVmax, carried prognostic significance [11]. Similarly, Ohlendorf et al. evaluated 32 metastatic GEP-NET patients and found that whole-body SSR-derived tumor volume and total lesion SSR were associated with chromogranin A and shorter progression-free survival, with high whole-body tumor burden associated with worse outcome (HR 5.16) [12]. Taken together, these studies indicate that receptor-based PET metrics are clinically meaningful, but they mostly address tumor burden quantification before treatment, whereas our study addresses the different and more pragmatic question of whether treatment effect can be captured on paired pre/post imaging using simple semiquantitative variables. This concept was also visually supported by the quadrant distribution of paired molecular and morphologic changes, where the largest subgroup consisted of patients showing TBRmax reduction without concomitant lesion shrinkage on contrast-enhanced CT (Fig. 2).

A particularly relevant aspect of our results is the apparent value of normalized PET metrics. In our cohort, TLRmax and especially TBRmax showed more robust paired changes than lesion size and identified a sizeable group of patients with molecular improvement without concomitant CT shrinkage. This is in line with the lesion-based study by Aouf et al., in which baseline SUVmax and SUVmean were poorly correlated with lesion-based functional response after PRRT, whereas tumor-to-liver ratios were significantly associated with response [7]. It is also consistent with the more recent RECIN framework proposed by Satapathy et al. in a post-hoc analysis of 72 patients from the LuCAP trial, where only 23/72 patients (32%) achieved RECIST partial response, but 40/72 (56%) fulfilled PET-based RECIN partial response; notably, 17 of 42 patients (40%) categorized as RECIST stable disease were reclassified as RECIN partial response, and RECIN showed better progression-free survival discrimination than RECIST (HR 0.33 vs. 0.52) [13]. Although our study does not propose a new formal response system, these data strongly support the same underlying concept: receptor-based molecular change may be more informative than simple morphologic size reduction in indolent NETs treated with PRRT. Our results also complement more complex imaging and biomarker models without competing directly with them. In the prospective phase II LUMEN study, multimodality imaging, dosimetry, and early treatment-related changes were integrated to predict PRRT outcome [6]. Parallel LUMEN analyses further showed that tumor absorbed doses may evolve heterogeneously over treatment cycles, with tumor absorbed doses decreasing by approximately 13% per cycle in pancreatic NETs and kidney activity showing the opposite trend, thereby supporting the rationale for personalized dosimetry rather than fixed assumptions [14]. In addition, in the organ-at-risk analysis from the same prospective cohort, the most common adverse events were anemia and lymphopenia (65%), grade ≥ 3 lymphopenia occurred in 43%, median isotopic GFR declined by 11%, yet no clear association was found between kidney, bone marrow, or spleen dosimetry and toxicity [15]. These reports underscore how sophisticated response and toxicity modeling is increasingly feasible, but they also highlight the gap our study addresses: most centers still need simple, reproducible PET-derived markers that can be extracted from routine scans without whole-body segmentation, serial dosimetry, or composite models. The same pragmatic perspective applies when our results are compared with real-world PRRT series. Casas et al. analyzed 36 metastatic NET patients treated with ^177Lu-DOTATATE and found that outcome was influenced by tumor grade, bone metastases, and mismatch between Octreoscan and CT findings before PRRT; in multivariable analysis, high Ki-67, discordant pre-treatment imaging findings, and previous treatment-related toxicity were adverse factors [16]. In a prospective Japanese phase 1/2 study including 15 advanced NET patients, Kudo et al. reported an objective response rate of 53%, a 52-week progression-free survival rate of 80%, and grade ≥ 3 lymphopenia in 47% of patients [17]. More recently, De Silva et al. showed in 105 patients that low baseline FDG-PET metabolic tumor volume or total lesion glycolysis was associated with longer progression-free survival after PRRT (45.7 vs. 21.6 months; HR 0.35), again indicating that functional imaging contains clinically relevant information not captured by morphology alone [18]. Thus, while the literature consistently supports the value of imaging-derived biomarkers in PRRT, it also shows that the field remains fragmented across different tracers, endpoints, and analytic frameworks. Our study contributes a narrower but clinically usable message within this landscape. Another important point is that much of the contemporary PRRT literature has shifted toward selection, safety, and treatment personalization, rather than straightforward post-treatment receptor-PET response assessment. Heckert et al. reported that in a U.S. cohort of 55 patients scheduled for PRRT, 11/52 who started treatment discontinued it early, and pretreatment liver function test abnormalities were strongly associated with treatment cancellation or discontinuation (odds ratio 12.0) [19]. In a Mayo Clinic analysis of 371 PRRT-treated patients, Gococo-Benore et al. specifically examined the subgroup with more than 75% liver involvement and found only one case of hepatotoxicity among 15 such patients, with no grade 3–5 liver failure events [20]. In a much larger retrospective cohort of 557 NET patients, Papantoniou et al. showed that lower albumin and higher C-reactive protein (CRP)-based indices, but not derived neutrophil-to-lymphocyte ratio (dNLR), were independently associated with shorter overall survival, adding further support to the idea that PRRT outcome is shaped by systemic and host-related factors as well as by tumor imaging features [21]. In a smaller imaging-linked cohort, Ohlendorf et al. also found that CRP, absolute neutrophil count (ANC), and platelet×CRP multiplier were higher in non-responders and that CRP and neutrophil-to-lymphocyte ratio (NLR) were associated with change in whole-body tumor burden after two PRRT cycles [22]. These studies are important, but they also show that the literature is increasingly moving toward multidimensional prediction models. In that setting, our study deliberately remains simpler: it asks whether paired semiquantitative receptor-PET metrics can make post-treatment change more visible than lesion diameter alone.

The present study has several limitations. First, it is retrospective and includes a relatively small cohort, which limits statistical power and precludes robust survival modeling. Second, our study was based on an exploratory paired target-lesion analysis derived from the institutional database. According to the institutional workflow, the target lesion selected at baseline was the lesion with the highest SUVmax and was then reassessed after PRRT. However, because lesion descriptors were not fully standardized across time points, exact lesion-by-lesion retrospective verification could not be confirmed in all cases from the database. Accordingly, the observed paired changes should not be interpreted as those of a formally adjudicated lesion-matched longitudinal analysis, and some residual selection or classification bias related to the retrospective dataset cannot be excluded. Third, although lesion size was measured on the contrast-enhanced CT component, the morphologic comparator used here should not be interpreted as formal RECIST 1.1 assessment. Fourth, semiquantitative measurements were extracted from the institutional dataset rather than from a centralized rereading workflow. Finally, the final response categories available in the clinical database were useful for exploratory contextualization but were not designed as a primary endpoint. These limitations are relevant and should prevent overinterpretation of the data [3, 13].

Despite these caveats, the message of the study is consistent and, in our opinion, clinically useful. We are not proposing that simple paired PET metrics replace RECIST, whole-body volumetric analyses, FDG-based phenotyping, or personalized dosimetry. Rather, our data suggest that semiquantitative SSTR PET may provide complementary information on treatment-related change beyond single-lesion size alone in this exploratory paired target-lesion framework, and that normalized metrics—particularly liver- and blood-normalized ratios—deserve attention as practical biomarkers of response. In everyday post-PRRT follow-up, clinicians frequently face lesions that remain morphologically stable despite a visibly lower receptor signal. Our results suggest that this discrepancy is not merely anecdotal, but measurable.

In conclusion, this exploratory paired target-lesion analysis suggests that semiquantitative SSTR PET-derived parameters, particularly normalized uptake ratios, identify treatment-related changes after 177Lu-DOTATATE and may provide complementary information beyond single-lesion size assessment. These results should be interpreted cautiously, but they support further investigation of simple PET-based metrics as practical tools for post-PRRT assessment in GEP-NENs.
